# Institutional Capacity for Science Diplomacy in Central America

**DOI:** 10.3389/frma.2021.663827

**Published:** 2021-07-14

**Authors:** Maria Esteli Jarquin-Solis, Jean-Christophe Mauduit

**Affiliations:** ^1^ School of Political Sciences, University of Costa Rica, San José, Costa Rica; ^2^ Department of Science, Technology, Engineering and Public Policy, University College London, London, United Kingdom

**Keywords:** science diplomacy, institutional capacities, international cooperation, central America, foreign policy

## Abstract

Science, technology, and innovation (STI) is increasingly gaining in importance on the foreign policy agenda of governments worldwide. However, the implementation of science diplomacy strategies requires STI institutional capacity and strong interfaces with policy and diplomacy. This research first maps the STI public institutions of the six member countries of the Central American Integration System (Guatemala, El Salvador, Honduras, Nicaragua, Costa Rica and Panama) and then draws their capacity to connect internationally in order to highlight their potential for science diplomacy. Variables such as the year of creation and mandates of scientific councils, secretariats, national academies, international cooperation departments and ministries are analyzed. The study reveals several public management challenges stemming from the institutional disparity and complexity of the region, already marked by significant asymmetries of human development between the various countries. Highlighting and understanding such challenges may be helpful for countries in the region in developing meaningful strategies around science diplomacy.

## Introduction

Climate change, infectious diseases, and food scarcity are amongst the many challenges that Central America is currently facing. Although science, technology and innovation (STI) issues are increasingly gaining in importance on the agenda of governments, different institutional and geopolitical integration challenges persist. Beyond the deployment of appropriate national STI policies in Central America (Lemarchand, 2010), responding to these complex issues will also require properly integrating science and foreign policy, leveraging scientific networks internationally and a coordinated response between countries in the region.

Science diplomacy is a relatively new field of scholarly study that focuses on these issues. While it has been pointed out that its exact aims, scope and stakeholders are still ill-defined (E.g. Kaltofen and Acuto 2018; Legrand and Stone 2018; Flink and Rüffin 2019; Ruffini 2020 and references therein) and that it is sometimes used as a convenient solutionist narrative (Rungius and Flink, 2020), science diplomacy has been taken up by advanced economies and scrutinized by scholars (E.g. Flink and Schreiterer, 2010; Ruffini 2017, Krasnyak, 2018; Ruffini 2021) and has gained ground in some of the BRICS countries (E.g., Pandor 2012; Oliveira Anunciato and Marques Sá dos Santos, 2020; Griset 2020). Despite the growing interest for science diplomacy in Central America (e.g., Panama), little academic attention has been devoted to the region and in particular its respective national institutions and their capacity to engage regionally and internationally (Gual Soler, 2014).

When focusing on science diplomacy as driven by the state, one of the taxonomies (Gluckman et al., 2017) proposes that it seeks to advance a country’s national needs, address cross-border interests and to meet global needs and challenges. States use a variety of institutions to engage along these broad lines, sometimes in a concerted manner that could be construed as a science diplomacy “strategy”^1^ (E.g., Ruffini, 2021). Since priorities may vary from state to state, no single science diplomacy strategy can be identified: these depend on their cultural and political context (Flink and Schreiterer, 2010; Krasnyak, 2018; Epping, 2020). In many cases, states have yet to actively engage in science diplomacy (whether through a strategy formulated in advance or in an ad-hoc manner) or to recognize the potential of its institutions to do so. In some instances, they may simply not have the institutional capacity.

While the stakeholders responsible for the strategy vary from country to country (E.g., the Ministry of Foreign Affairs or MoFA, an inter-ministerial task forces of its MoFA and Ministry of Science and Technology or sometimes a mix of institutions, etc.,), it nonetheless relies on the capacity of the state to mobilize its ecosystem through its own national institutions. In order to start formulating a strategy for science diplomacy, it is therefore necessary for states to take stock of their institutions, their inter-linkages and interfaces between science and diplomacy. While research is still lacking on what can be considered institutional capacity for science diplomacy, it is clear that having national institutions that are already engaging internationally in science is a helpful starting point. In this paper, we therefore carry a first landscape analysis of the STI publicly-funded institutions in Central America and their potential to engage in science diplomacy, identifying challenges and opportunities specific to the region.

## Research Objectives and Methods

The purpose of this research is to analyze the institutional capacity for science diplomacy of the six original (1991) member countries of the Central American Integration System (SICA): Guatemala, El Salvador, Honduras, Nicaragua, Costa Rica and Panama. We first mapped these countries’ STI public institutions, and then looked at their capacity to connect internationally as a means to highlight their *potential* for science diplomacy.

According to Elorza et al. (2020), the most common objectives of science diplomacy strategies are: “1) strengthening bilateral scientific collaborations and the support of countries’ STI interests, 2) facilitating evidence-informed positions of the country in multilateral endeavours and global challenges, 3) bringing new scientific opportunities and scientific talent to the country, 4) using scientific collaborations as a tool for improving bilateral relations with strategic countries, 5) acknowledging STI as a key asset of the country in its image abroad, 6) facilitating country companies to have a good place in the international innovation market as well as in the research and development international arena”. This research is restricted only to a subset of these objectives and focuses on objective one (and by proxy, objectives four and five) by looking at ministries and on objective two by looking at scientific councils and national academies. It is however important to note that objective one needs to be “coordinated” institutionally to be construed as part of science diplomacy (Krasnyak and Ruffini, 2020). This paper considers the *potential* for these institutions to align with the nations’ foreign policy, not whether they are engaging in science diplomacy already.

The state can leverage a host of stakeholders for science diplomacy, from its own ministries to universities, the private sector, its scientific community at home or diaspora overseas, etc., maximizing and aligning their actions with the state’s perceived needs in engaging in science at the international level. However we choose here to restrict the scope of our analysis to public institutions. The unit of analysis of this research is therefore “STI national institutions”, meaning (publicly-funded) national and centralized agencies that provide scientific and technological services in Central America. Institutions analyzed here are therefore ministries, scientific councils and most national academies.

It is also worthy to note that while science encompasses natural sciences, engineering and social sciences, here we choose to restrict our analysis to the first two. In addition, STI here covers the realm of basic sciences to the applied sciences and innovation derived from natural sciences and engineering (while noting that the innovation process should not be construed as linear one and that social sciences also contribute). Nonetheless, this means that we choose to restrict our subsequent analysis to the following sectors: science (as a general denomination), health, agriculture, energy, environment and education (the latter is partly focused on STI), leaving aside social issues such as housing, infrastructure, transport, economy, commerce, development and industry. It is however necessary to mention that part of the STI spectrum is therefore missing from this initial analysis.

The research process was developed in five phases. First, each of the government ministries which provide a scientific or technological service was identified for every SICA country. This includes looking at ministries working on health, agriculture, energy, environment and education, and those labelled as more broadly focusing on “science and technology”. Second, the organization chart of each of these government agencies was reviewed (using their official websites, available documentation, academic and grey literature) to identify international cooperation departments within them. Then, we identified all the scientific councils and national academies of the region through grey literature and websites of various organizations such as the “Inter-American Network of Academies of Science” (IANAS)^2^ or the “InterAcademy Partnership” (IAP)^3^.

The fourth stage was to analyze the mandate of these STI national institutions, with an emphasis in their roles and responsibilities towards internationalization. Finally, a database was built to organize key information on all these institutions which includes the following eight variables: 1) the name of the entity, 2) the SICA country to which it belongs, 3) the type of institution (a ministry, a scientific council or a national academy), 4) its acronym, 5) year of creation, 6) thematic focus, 7) a list of its international cooperation units when available, 8) the broad mandate of the entity. The database lists 45 entities, including 33 ministries, eight scientific councils, and four national academies.

This is the first inventory that analyses the range of actions of these institutions as potential science diplomacy actors in the region. However, the study has several limitations. First, this mapping does not include local governments, decentralized government entities or other stakeholders in Central America which could be important for their scientific and technological international engagement (E.g., higher education institutions, private sector or organized civil society). More importantly, this simple inventory of institutions and mandates does not address whether they are working well, if coordination is effective nor how their activities are perceived internationally.

## Results

The first step was a mapping of the main STI publicly-funded institutions in the SICA region (here encompasses ministries, scientific councils and national academies) and the second to analyze their mandates and structures to engage with the international realm.

**FIGURE 1:**
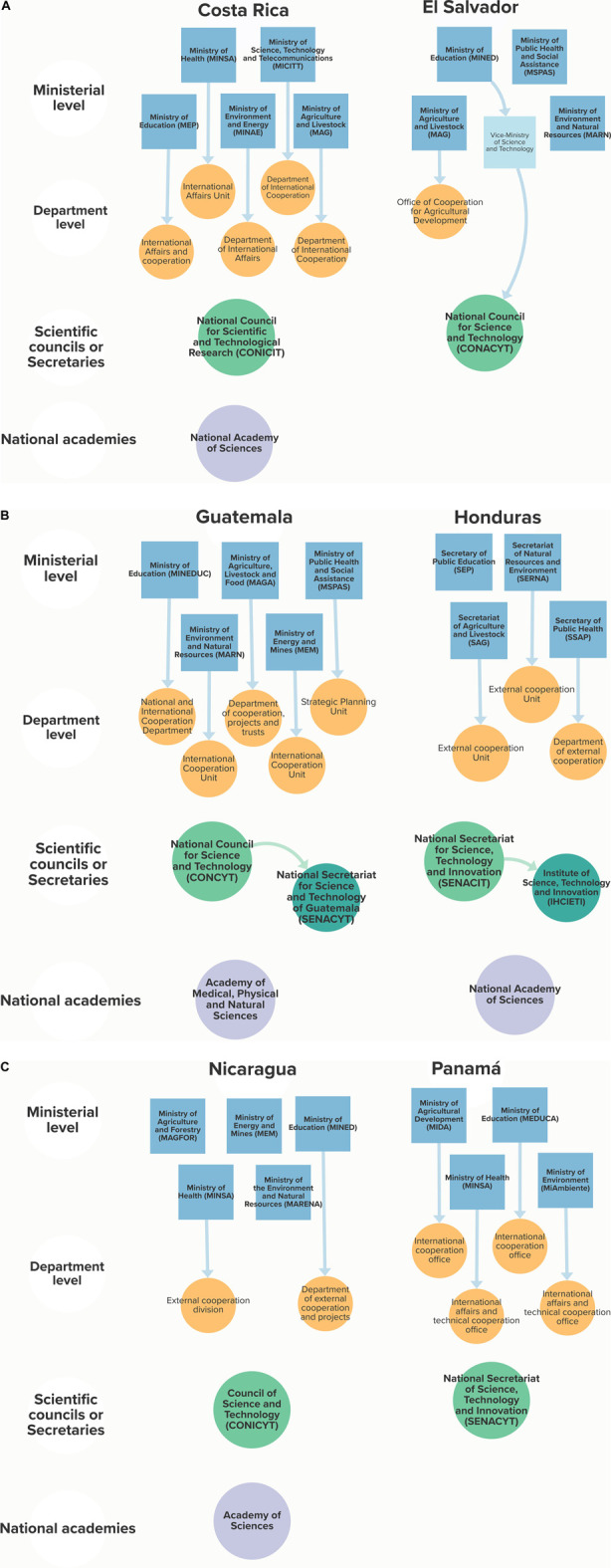
A mapping of the institutional capacities for science diplomacy in the six SICA countries (2021), arranged by alphabetical order (from left to right and top to bottom). The figure shows the ministries and vice-ministries in blue (first row) and their international cooperation departments in orange (second row), the scientific councils or secretaries in green (third row) and the national academies in purple (fourth row). Note that some entities may be missing due to specific definitions of ‘STI institutions’ (see *Research objectives and methods*).

It is useful to first (re)-take stock of these institutions in their national context and do a short comparative analysis across the region. Indeed, highlighting similarities (or differences) across national STI ecosystems may provide some useful clues for the capacity for national science diplomacy strategies, cross-border collaboration along similar STI themes or the development of concerted regional foreign policies in STI. Figure 1 summarizes the different actors identified in this research at the ministerial and departmental level (respectively in blue and orange), as well as the scientific councils (in green) and national academies (in purple) of the SICA region.

### STI-Related Ministries

There are 27 government ministries (featured in blue in Figure 1) with an STI focus (as defined in *Research Objectives and Methods*) in the SICA countries. These national agencies address issues of science, health, agriculture, energy, environment, and education. The institutional design in each of these topics varies from country to country. The most striking feature of the region’s government institutions is that Costa Rica is the only country with a dedicated Ministry of Science and Technology (MICITT, created in 1990). Another peculiarity is that El Salvador created a Vice-Ministry of Science and Technology within its Ministry of Education in 2009. According to an analysis of their mandates, both have similar functions (since they are the governing body in STI issues): they are responsible for coordinating the “national STI ecosystem”, which includes the design of STI policies^4^.

Looking at the year of creation and mandates of these 27 institutions, a noticeable common feature shared by the region is that these were created in two main waves of institutional experimentation over time. The first wave included the education, agriculture, and health portfolios, which were implemented from the second half of the 19th century until about 1950. By that time, the entire region already had ministries covering these topics. In most cases, the other ministries (environment, energy, and science) were then part of a second wave of institutional innovation that mostly occurred after 1990. For example, most SICA countries created ministries with an environmental focus after 1990 (with the exception of Honduras’ “SERNA”, created in 1954). Ministries dealing with the energy sector were also created during this second wave, such as Costa Rica (1990) and Nicaragua (2007), although Guatemala did so slightly before (1983). This second wave of creation of ministerial institutions is interestingly correlated with the birth of other STI-relevant institutions, the scientific councils, which we focus on in subsection b) below.

The second dimension of this analysis then focused on the international outlook of these ministries. If Central American governments are using science and technology as a tool to respond to global challenges, what institutional capacity do they have to engage in international scientific collaboration and (government-driven) science diplomacy? As a proxy, the research identified the international affairs departments within the STI ministries as a potential building block for a science diplomacy portfolio. In addition, international cooperation has been considered a key instrument in building capacities in science and technology in Central America (Bonilla, 2018).

As shown in Figure 1, there are 20 international affairs units (in orange) within the STI ministries (in blue) analyzed in the section above. The number of such units is uneven from country to country. All the ministries (as defined in *Research Objectives and Methods*) of Costa Rica, Guatemala and Panamá have an international cooperation unit, followed by Honduras (to the exception of SEP, Figure 1). In Nicaragua only two ministries (health and education) have such a unit and in El Salvador only one does (agriculture). The approach also differs widely from one country to another: the portfolios that these units may be tasked with are disparate across the region, which may present a challenge for regional coordination. Most seem to have been created in the 21st century, except for the case of the department at the Costa Rican ministry of education (1982) and the one within the ministry of agriculture in Panamá (1990). Finally, the names of these units vary considerably even within the same country: for example, in the case of the MSPAS in Guatemala, its “strategic planning unit” is in charge of the international cooperation of that ministry.

This landscape analysis of several STI-focused ministries and their institutional capacity to engage with the international realm through their international affairs units is only a first step, however. A crucial next step would be to investigate their links and overall policy alignment to their countries’ MoFA—or other ministries that could be equally important in designing a strategy (in Costa Rica for example, the Ministry of National Planning and Economic Policy coordinates the country’s international cooperation). This would require interviews, surveys or specific field work and is beyond the scope of this paper.

### Scientific Councils

In this next subsection, we focus on the scientific councils in SICA, taking a closer look at their year of creation, mandates, institutional arrangements and linkages. These, alongside national academies (see *National academies*) can play a role in fulfilling parts of a science diplomacy strategy.

As can be seen in Figure 1, all SICA countries have scientific councils or secretaries for STI. Most were created in the 1990s, with the exception of Costa Rica and its National Council for Scientific and Technological Research (CONICIT), established nearly 2 decades earlier in 1972. The institutional arrangement of these scientific councils has evolved significantly over time, resulting in a complex picture which is not captured by Figure 1.

The case of the Honduran Council of Science and Technology (COHCIT) is a clear illustration of this complexity. The Council was created in 1993 and then replaced in 2013 by two entities: 1) the National Secretariat for Science, Technology, and Innovation (SENACIT) and 2) the Institute of Science, Technology, and Innovation (IHCIETI). Both are an integral part of the National System of Science, Technology, and Innovation of Honduras^5^. This transformation was originated by the law for the promotion of scientific and technological development passed in 2013 (No. 276-2013). It is worth noting that the relationship between these two entities was only clarified a few years later in 2020, following the publication of a new regulation (No. 047-2020). An analysis of the mandates showed that SENACIT oversees the promotion of policies pertaining to the scientific and innovation development in Honduras and coordinates the different stakeholders. The IHCIETI is the technical agency responsible for the design and implementation of strategies, policies and programs for scientific research, and technological development, such as a national plan for STI through which it will establish the country’s priorities on the topic.

Quite a few of the STI systems in SICA follow this two-pronged approach. Guatemala is another example: its National Council for Science and Technology (CONCYT) formulates the STI policy and its associated budget (as well as approves international technical cooperation) whereas its National Secretariat for Science and Technology (SENACYT) executes and implements the policy decisions of the former. El Salvador recently created two key institutions in its STI ecosystem, establishing a Vice-Ministry of Science and Technology in 2009 and a National Council for Science and Technology (CONACYT) in 2013, both attached to the Ministry of Education. In this case, a much more specific role can be identified at the ministerial level since its objective is to develop an information and communication technology policy for schools in the country. The Council executes national policies on scientific and technological development and encourages innovation.

As explained above, Costa Rica has a slightly different arrangement, in that it involves a ministry and a scientific council, yet the similar two-headed structure remains. MICITT generates and promotes compliance with public policies on science, innovation, and technology while the National Council (CONICIT) executes policies and finances research and development.

A detailed analysis of the mandates revealed that each country carries out different activities for the international engagement of its scientific councils. El Salvador’s CONACYT focuses on promoting research, technology transfer and R&D through international alliances. Panama’s SENACYT centers their attention on strengthening global cooperation, through signing agreements with international organizations. Guatemala created a national scientific and technological information system to facilitate relations with international networks. CONICIT (Costa Rica) focuses on financing scholarships abroad and IHCIETI (Honduras) seeks financing for the National Plan for Science, Technology, and Innovation.

### National Academies

National academies are also an important actor in the STI ecosystems which usually finances all or parts of their operations through public funding. They play a critical role at the national level but also internationally (e.g., through the informal connections of their members or formal connections to overseas networks) and may play a role in informing the science diplomacy agenda of their country^6^, as is the case in the United States^7^ or South Africa (Maphosa, 2019) for example.

Four of the SICA countries have official national academies (Costa Rica, Guatemala, Honduras and Nicaragua). Neither El Salvador nor Panamá have national academies (though for the latter its “Panamanian Association for the Advancement of Science” seems to be acting as a substitute), which may represent an important gap for the coordination of the scientific community and research activities (both domestic and international) of these countries.

As can be seen in Table 1, these national academies have been created at different times in history, from 1945 (Guatemala) all the way to 2005 (Nicaragua). This research also found important differences from country to country in terms of funding and mandates, among others.

**TABLE 1:** Year of creation and sources of financing of National Academies in Central America.

	Costa Rica	El Salvador	Guatemala	Honduras	Nicaragua	Panamá
National Academy	National Academy of Sciences	None	Academy of Medical, Physical and Natural Sciences	National Academy of Sciences	Academy of Sciences^a^	None
Year of creation	1992	*Not applicable*	1945	1983	2005	*Not applicable*
Source of financing	Central Government	*Not applicable*	Public University	Central Government	Nonprofit Civil Association	*Not applicable*

a Note that Nicaragua's Academy of Sciences is a nonprofit and not publicly funded, but it is included here for reference

In terms of sources of funding, according to documents and budgetary information publicly available on these various entities’ websites, the Costa Rican academy is financed directly by the Ministry of Science and Technology (MICITT), while the Honduran one is sponsored by a Presidential Department attached to the Secretariat of General Coordination of the Government (SCGG), providing it with a strategic location for the provision of scientific advice in the decision-making process of the country. On the other hand, Guatemala’s national academy was created by a public university (San Carlos University) and in Nicaragua, it is a non-profit civil association.

The statutes of the national academies of Costa Rica, Guatemala, and Honduras include establishing cross-border scientific collaborations through agreements with foreign institutions, as well as organizing and participating in international scientific conferences and forums to position their countries on the international scientific scene. Whether this was done in direct consultation with the Ministry of Foreign Affairs of their respective country is beyond the scope of this paper but would be a valuable next step. Gual Soler (2020) identified that the Costa Rican MoFA and the National Academy of Sciences have articulated efforts to link diplomatic practice to local research around natural disaster prevention, biotechnological development, amongst others.

All SICA national academies (with the obvious exception of El Salvador) are members of the regional Inter-American Network of Academies of Science. Guatemala, Honduras and Nicaragua are members of the InterAcademy Partnership (IAP), an international network of more than 140 academies of science, medicine and engineering from around the world^8^. Surprisingly, Costa Rica is not a member of IAP.

### A Dichotomy Between Diplomacy and Science

**FIGURE 2:**
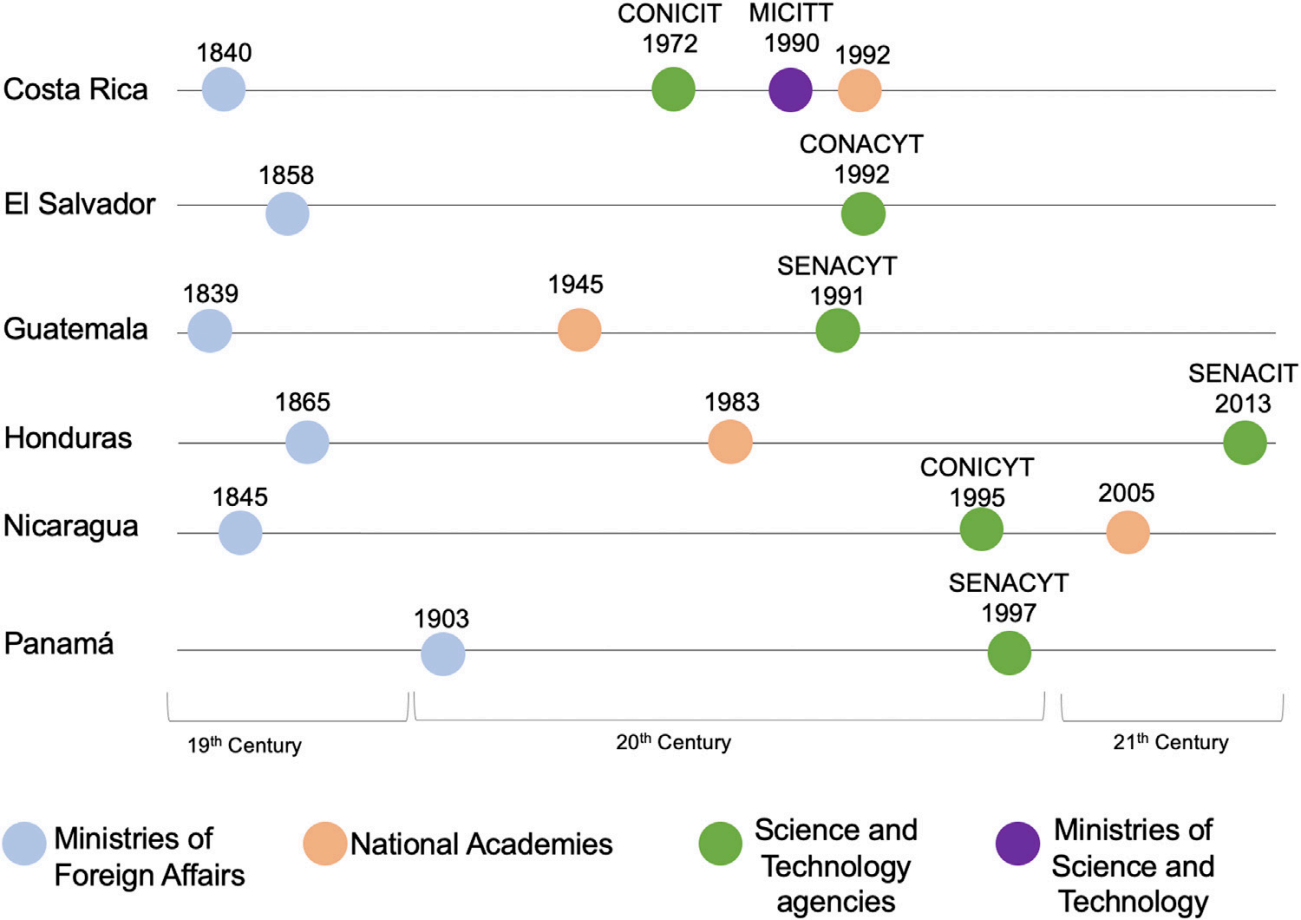
This timeline shows the dates of creation of the six SICA countries’ Ministries of Foreign Affairs (in blue) and Science and Technology (in purple) as well as their respective S&T agencies (in green) and national academies (in orange). It only depicts the current institutional setting and hence does not include agencies that have been removed or replaced over time. In addition, other ministries which have a portfolio connected to scientific issues (see *Results*) are not featured here so as not to overcrowd the timeline.

In order to contextualize the evolution of science and diplomacy institutions in the region, Figure 2 visualizes the year in which these agencies were created over time. As can be seen in the Figure, the Ministry of Foreign Affairs were mostly created in the 19th century or at the turn of the 20th century, whereas the national STI institutional infrastructure in Central America is relatively recent. The creation of these STI national bodies and institutions coincides with the period of greatest institutional innovation in the history of the region, but also with the peace and post-conflict processes experienced in the eighties and nineties (Vargas-Cullell and Duran, 2016).

Beyond the timeline, Figure 2 provides an interesting overview of the SICA countries national institutions and further highlights the difference in the institutional hierarchy of agencies between the two realms of science and diplomacy. Diplomatic matters are always led by ministries, (which represent the highest hierarchical level of the public administration), while overarching (non-thematic) STI issues do not fall within the remit of a single coordinating ministry (with the exception of Costa Rica) but rather within secretariats, scientific councils and national academies (or are fragmented within various thematic ministries). These national agencies, while in charge of overarching STI issues, do not necessarily carry the same political and institutional weight as ministries.

This could result in an institutional “imbalance” when setting a science diplomacy agenda in collaboration with other government ministries, as scientific councils may not participate in cabinet meetings, hence their access to have the necessary communication and coordination with each of the governing ministers of the different sectors is more limited. Finally, they do not have direct access to multilateral and international forums the MoFA and other ministries usually have under their legal mandate.

This may have resulted in science still playing a limited role in the foreign policy of the region. However, it is worth noting however that the lack of an STI ministry may not be an impediment to launch strategies around science diplomacy, as Panamá demonstrated in2018^9^ when it became the first Latin American country to define a national strategy promoted by the MoFA and SENACYT (Gual Soler, 2020).

## Discussion: Towards a Science Diplomacy Portfolio in Central America

This exploratory research carried out a first diagnosis of the current STI institutional landscape in the SICA region, and the potential for these institutions to engage in science diplomacy. It reveals various challenges at the national and regional level, stemming from the multiplicity and disparity of institutions, and the heterogeneity of their mandates across Central America. Understanding such challenges may be helpful for countries in the region in developing meaningful strategies around science diplomacy.

The institutional landscape analysis shows that most STI national bodies and institutions in the SICA countries were created during the period of greatest institutional innovation in the region. However, this also led to a multiplicity of entities and fragmentation of roles. This may be detrimental to achieving an effective science diplomacy strategy unless complex inter-linkages are constructed via inter-ministerial tasks forces or other processes. Another challenge is the gap that exists in the institutional hierarchy of agencies between the two realms of science and diplomacy. Diplomatic matters are always led by ministries, while STI issues are fragmented in various thematic ministries and the main overarching STI portfolio is typically led by scientific councils, pointing to a potential institutional imbalance^10^. This would imply better clarifying current roles and competencies. A future study could look in more detail at interlinkages across these multiple actors to better understand how they may play a role in setting the science diplomacy agenda of the country in order to advance the countries’ national needs and interests, as well as strengthen bilateral scientific collaborations (S4D4C objective 1). It would also be key to identify if and when coordination with the MoFA may play a multiplying role, especially to facilitate evidence-informed positions of the country in multilateral endeavours and global challenges (S4D4C objective 2).

A detailed analysis on the scientific council’s mandates also reveals that each country carries out different activities for the international engagement of these entities. These institutions present a wide diversity in their structures and mandates across the region, which could make it more difficult for the SICA countries to address their cross-border interests or to strengthen bilateral or multilateral collaborations in STI. In addition, at a regional level, it may also impact the ability for the SICA countries to design consistent and complementary regional policies that address common STI-related challenges. This may be an opportunity to better integrate these institutions in the regional agenda, as a means to improve bilateral relations (S4D4C objective 4), address cross-border interests and better project the region internationally in STI (S4D4C objective 5).

Despite these challenges, science diplomacy represents an opportunity for countries in Central America and for the region as a whole. Future research is crucially needed to better define the necessary institutional capacity and cross-institutional linkages required for the deployment of a successful science diplomacy strategy. This is particularly salient in the context of emerging countries in light of some of their institutional challenges. Moreover, in-depth case studies of the SICA countries will be necessary to explore whether their respective institutions are working as intended to enable the integration of science within foreign policy agendas, if coordination is effective and how their activities are perceived internationally. This would require dedicated interviews, surveys, and ethnographic field work. Going beyond the state’s institutions, it would also be key to investigate the role of non-governmental and decentralized institutions in the region for science diplomacy, such as local governments, universities, nonprofits and the private sector.

## Data Availability

The raw data supporting the conclusion of this article will be made available by the authors, without undue reservation.

## Footnotes

1 Rapport, “Une diplomatie scientifique pour la France”, Ministere des Affaires Etrangeres, 2013, https://www.diplomatie.gouv.fr/IMG/pdf/Rapport_Complet_DiplomatieScientifique_2013_cle8a68fb.pdf

2
https://ianas.org/

3
https://www.interacademies.org/

4 According to Law No. 7169 (*Law for the Promotion of Scientific and Technological Development* of Costa Rica) and from Decree No. 234 (*Law of Scientific and Technological Development* of El Salvador).

5 Together with the National Council for the Promotion of Science, Technology, and Innovation (CONFOCIT) and the National Fund for Financing Science, Technology, and Innovation (FONAFICIT).

6
https://www8.nationalacademies.org/pa/projectview.aspx?key=51726

7 Diplomacy for the 21st Century: Embedding a Culture of Science and Technology Throughout the Department of State. Washington, D.C.: National Academies Press, 2015.https://doi.org/10.17226/21730.

8
https://www.interacademies.org/network/member-academies

9 EU Science Diplomacy. “Panama’s Science Diplomacy Strategy: Current State and Future Challenges,” June 29, 2020. https://www.s4d4c.eu/panamas-science-diplomacy-strategy-current-state-and-future-challenges/.

10 It should be noted however that Panamá´s science diplomacy strategy was launched in 2018 in spite of this particular challenge.
